# Associations between socioeconomic status and pregnancy outcomes: a greater magnitude of inequalities in perinatal health in Montreal than in Brussels

**DOI:** 10.1186/s12889-022-13165-1

**Published:** 2022-04-25

**Authors:** Mouctar Sow, Marie-France Raynault, Myriam De Spiegelaere

**Affiliations:** 1 School of Public Health, University of Montreal, Quebec, Canada; 2 Université Libre de Bruxelles, École de santé publique, Brussels, Belgium; 3 Lea-Roback Research Centre on Social Inequalities in Health, CRCHUM, Quebec, Canada

**Keywords:** Social inequities in health, Inequalities in perinatal health, Poverty, Income inequality, Low birth weight, Preterm birth, Pregnancy outcomes, Immigration, Comparative study

## Abstract

**Background:**

Comparing health inequalities between countries helps us to highlight some factors specific to each context that contribute to these inequalities, thus contributing to the identification of courses of action likely to reduce them. This paper compares the associations between socioeconomic status (SES) and 1) low birth weight (LBW) and 2) preterm birth, in Brussels and Montreal (in general population, natives-born mothers, and immigrant mothers).

**Methods:**

A population-based study examining associations between SES and pregnancy outcomes was conducted in each city, using administrative databases from Belgian and Quebec birth records (*N* = 97,844 and 214,620 births in Brussels and Montreal, respectively). Logistic regression models were developed in order to estimate the relationship between SES (maternal education and income quintile) and pregnancy outcomes, in each region. The analyses were first carried out for all births, then stratified according to the mother’s origin.

**Results:**

For the general population, SES is associated with LBW and preterm birth in both regions, except for income and preterm birth in Brussels. The association is stronger for mothers born in Belgium and Canada than for those born abroad. The main difference between the two regions concerns the magnitude of inequalities in perintal health, which is greater in Montreal than in Brussels among the general population. For native-born mothers, the magnitude of inequalities in perinatal health is also greater for mothers born in Canada than for those born in Belgium, except for the association between income and preterm birth. The socioeconomic gradient in perinatal health is less marked among immigrant mothers than native mothers.

**Conclusion:**

Significant differences in inequalities in perinatal health are observed between Brussels and Montreal. These differences can be explained by : on the one hand, the existence of greater social inequalities in Montreal than in Brussels and, on the other hand, the lower vulnerability of immigrants with low SES in Brussels. Future studies seeking to understand the mechanisms that lead to inequalities in health in different contexts should take into account a comparison of immigration and poverty contexts, as well as the public policies related to these factors.

## Background

Health inequities occur as early as the prenatal period and during the early years of life of the child [1–3]. Policies that influence the (re) production of social stratification (e.g. social policies, labour market integration policies) and reduce exposure to risk factors for disease, such as poverty, can have a positive impact on the health of the most vulnerable groups and contribute to the reduction of health inequities [4, 5]. During certain critical periods of life, such as pregnancy, the benefits of such policies may be even more important. Indeed, poverty before and during pregnancy (as well as the material and psychosocial consequences of low income) has a negative impact on the physical and mental health of the mother, which causes repercussions to the development of the foetus, and increases the risk of adverse pregnancy outcomes such as low birth weight or pre-term birth. Measures that improve household living conditions and children’s health as early as possible significantly contribute to breaking the vicious cycle of social inequalities in health [6–11].

Comparing health inequities from birth between countries or regions helps us understand the mechanisms specific to each context and identify courses of action likely to reduce such inequalities. Several articles compare health inequities in different contexts [7, 12–14]. Martinson and Reichman’s study [15], which compares the socioeconomic gradient with respect to LBW in the United States, Canada, Australia and Great Britain, is in keeping with this logic. The results showed a strong gradient in the USA when compared to the other countries.

This paper studies the relationship between socioeconomic status (SES) and two adverse pregnancy outcomes, low birth weight (LBW) and preterm birth, in both Brussels and Montreal. It identifies the main similarities and differences between these two regions and brings forth explanatory hypotheses for these observations. The analysis compares the scale of inequalities in perinatal health in the general population, much like Martinson and Reichman did [15].

In addition, it compares the patterns of these inequalities between mothers born in Belgium or Canada and immigrant mothers. Such a distinction is all the more relevant since epidemiological studies show that the association between SES and pregnancy outcomes varies not only according to the contexts and indicators considered, but also according to the population studied [7, 12, 16–18]. In fact, an important finding of epidemiological studies is that, while in the general population SES indicators are good predictors of prematurity, low birth weight and stunted growth, they are not always associated with these pregnancy outcomes in immigrant mothers. More precisely, in some immigrant groups, the risk of adverse pregnancy outcomes does not differ (or differs only slightly) according to the mother’s level of education. In particular, this lack of a socioeconomic gradient has been observed among Hispanic mothers living in the United States [12, 19]. This result is consistent with studies showing that this ethnic group, despite their socioeconomic disadvantage, has similar (and in some subgroups even lower) prevalence of adverse pregnancy outcomes to white American mothers [20–22]. This finding has been termed an epidemiological paradox. Similar patterns were found among Turkish and North African mothers living in Belgium; these groups have significantly lower prevalence of low birth weight and prematurity despite their marked socioeconomic disadvantage [18, 23, 24]. These findings highlight the importance of taking into account the effects of specificities and contexts linked to immigration, in particular by comparing the health gradient among different groups of immigrants to that observed among native-born women, in order to better understand the socioeconomic determinants of perinatal inequalities.

Our analysis focuses on two perinatal indicators: LBW and preterm birth, both of which are pregnancy outcomes that are strongly associated with SES [12]. They increase the risk of infant mortality and health problems in children and adults. We will compare inequalities in LBW and preterm birth in Brussels and Montreal. The latter are the largest cities of Belgium and Québec respectively, and they share sociodemographic similarities, particularly with respect to immigration. In fact, more than half of all births come from immigrant households in both regions [18, 25, 26]. The access to perinatal care is also comparable, with government health insurance plans and perinatal health prevention programs targeting vulnerable groups in both regions. However, social policies differ significantly between these two contexts, particularly with respect to minimum income protection measures, which are comparatively more generous in Belgium than in Quebec [10, 27].

This article studies the associations between socioeconomic status (SES) and 1) low birth weight (LBW) and 2) preterm birth, in Brussels and Montreal. Specifically, it compares the magnitude of inequalities in perinatal health between these two regions (in general population, natives-born mothers, and immigrant mothers).

## Methods

Two case studies were conducted. A study examining the association between SES and pregnancy outcomes was conducted in each city.

### Data sources

In Brussels, the data is based on singleton live births spanning from 2005 to 2010, which amount to 97,844. This data is the result of the combination of three administrative files: the birth register, containing the health data of newly born babies; the Crossroads Bank of Social Security (‘Banque Carrefour de la Sécurité Sociale’), which includes socioeconomic data on households; and the national register, which encloses data on migration. To our knowledge, this is the first study to combine these data in Belgium. For the administrative region of Montreal, the data comes from birth registers, and is based on 214,620 singleton live births that occurred between 2003 and 2012.

### Outcomes measures

This study focuses on two adverse pregnancy outcomes: low birth weight (LBW) and preterm birth. Low birth weight refers to a weight of less than 2500 g. Preterm birth refers to delivery before 37 weeks of gestational age. LBW and preterm birth are strongly associated with SES [12]. They increase the risk of health problems at birth and in childhood.

### Explanatory variables

#### Education

Maternal education was divided into three categories, taking into account the difference in school systems and diplomas in Belgium and Quebec. Mothers considered to have a high level of education are those who have obtained a university degree, or any kind of higher education degree in Belgium. This corresponds to who have completed at least 16 years of education in Belgium or Quebec. Mothers with less than 12 years of education are considered to be less educated: they did not graduate secondary school in Belgium or go beyond Secondary V in Quebec. Women who have completed at least 12 years of education but did not obtain a higher education degree are considered to have an intermediate level of education.

#### Income

Data from each region were considered. In Brussels, the data is based on households’ income and is derived from social security data [28]. These data comprise the yearly income from work and replacement income. They do not include income from real estate and movable sources. These are gross taxable annual incomes (after deduction of social contributions). In order to be able to compare households, these income data are based on household size, which is therefore a “household equivalent income” calculated by dividing the sum of monetary incomes received by each member of the household by the equivalent size of the household. This size is estimated by using the OECD-weighting scale. In the database, we have the equivalent household incomes by deciles, which are based on the income distribution for all Belgian households. This means that for any household that had a child during the study period, we are able to determine which income decile of the general population it falls into, but not its exact income level. The deciles have been grouped into quintiles. In this way, we can compare the perinatal indicators of Brussels children based on them belonging to one or other quintile in the general population.

The income data at the household level were not available for Quebec. Income data collected at the level of small geographic areas called dissemination areas were considered. These data were obtained through the national census conducted by Statistics Canada. In Quebec, census data are collected at several geographic levels, including the regional level. The dissemination area is the smallest geographic unit for which Statistics Canada releases census data [29]. Health inequalities are monitored at these different geographic levels [30, 31]. Given the limited availability of income data at the individual level for monitoring health inequalities, the question of using geographic data as a proxy for individual data arises. The relevant recommendations state that data obtained for the smallest geographical agglomerations, in this case dissemination areas, can represent the individual data. However, such use demands caution. This proxy may not be valid for areas where the socio-economic status of residents varies greatly, such as rural areas where postal codes cover large geographic areas. It is also not relevant for monitoring health inequalities in urban centres from a longitudinal perspective, as the neighbourhoods have a dynamic demographic composition. In général, geographic indicators are considered good proxies for individual situations when they relate to small, socio-demographically homogeneous agglomerations such as diffusion area in Montreal [30]. Therefore, we used the average income of the dissemination area as a proxy for the income of the families living there. The income data from the census file were integrated into the birth file by using the postal codes, which are available in both files. Each household was assigned the average income of its diffusion area. The variable was then categorised into quintiles according to the distribution of the study population. These quintiles are constructed on the population of mothers who gave birth during the study period, and therefore not on the general population, as is the case in Brussels.

### Statistical analysis

Two case studies were performed. A study investigating the association between SES and pregnancy outcomes was conducted in each city. Low birth weight and preterm delivery were analysed according to maternal education and household income. Logistic regression models were used to estimate crude and adjusted odds ratios of the associations between perinatal indicators and SES. The adjustment covariates were relationship status (being in a couple or not), maternal age, parity, and child sex. The analyses were first carried out for all births, then stratified by immigration status (native-born mothers vs immigrant mothers). Crude and adjusted ORs derived from the logistic regression and the *p*-value of the Wald test (with a significance level set at 5%) are presented. Analyses were processed through Stata, version13.

## Results

### Characteristics of births in Brussels and Montreal: important differences according to mother’s birthplace

There are on average around 16,300 singleton live births per year in Brussels and 21,500 in Montreal for the time periods studied. In both regions, more than half of the births were to foreign-born mothers. The distribution of SES according to the mother’s birthplace differs between Brussels and Montreal (Table 1). The percentage of less educated mothers is relatively higher in Brussels than in Montreal, whereas that of well-educated mothers is higher in Montreal than in Brussels. The difference between the two regions is even greater when comparing the situation of immigrant mothers. In Brussels, foreign-born mothers have lower income and lower education levels than those born in Belgium, while in Montreal the level of education is not correlated to maternal birthplace, and the income gap between immigrant mothers and Canadian-born mothers is less pronounced than in Brussels. The proportion of single mothers is higher in Brussels than in Montreal. The figures do not differ according to the mother’s birthplace for both regions.

**Table 1:** Characteristics of mothers and newborns in Brussels and Montreal

	BRUSSELS (2005–2010)			MONTREAL (2003–2012)		
	*Maternal birth place*			*Maternal birth place*		
	All Births	Born in Belgium	Immigrants	All Births	Born in Canada	Immigrants
N	97,844	39,591	55,333	214,620	97,520	112,468
% of births	100	40.46	56.55	100	45.4	52.4
**Maternal education (n)**	89,864	37,085	50,175	200,943	92,943	104,476
High (%)	31.66	40.64	24.66	46.14	47.23	45.30
Intermediate (%)	35.16	35.14	35.27	29.16	29.36	28.83
Low (%)	33.17	24.22	40.07	24.70	23.41	25.87
**Income Quintile (n)**	88,655	38,638	48,937	211,265	95,642	111,052
Top (%)	13.10	20.28	7.26	20.00	26.57	14.52
Fourth (%)	11.75	18.47	6.50	20.00	23.34	17.08
Midlle (%)	15.27	18.65	12.69	20.00	20.35	19.59
Second (%)	18.48	16.23	20.35	20.00	17.75	21.91
Bottom (%)	41.40	26.36	53.20	20.00	12.00	26.89
**Household situation (n)**	88,677	37,362	50,256	208,249	95,139	108,811
Lives alone (%)	16.16	16.40	15.91	9.94	10.18	9.69
**Maternal age (n)**	97,844	39,591	55,333	214,620	97,520	112,468
< 20 (%)	2.39	2.39	2.35	2.17	3.30	1.16
≥ 40 (%)	4.31	2.92	5.33	6.62	5.27	7.86
**Previous births (n)**	97,234	39,381	54,945	214,620	97,520	112,468
0 (%)	47.70	52.09	44.13	48.65	53.83	43.93
1–2 (%)	43.70	41.73	45.37	45.36	41.44	48.96
3 (%)	8.60	6.18	10.50	5.99	4.73	7.11
**LBW (n)**	97,844	39,381	55,333	214,589	97,509	112,463
%	4.64	5.08	4.31	4.49	4.32	4.65
**Preterm (n)**	95,490	38,670	54,009	214,587	97,509	112,462
%	5.22	5.48	5.02	5.82	5.72	5.95

### Associations between SES and adverse pregnancy outcomes

#### Greater inequalities in perinatal health in Montreal than in Brussels

In both regions, newborns of highly educated or high-income mothers are at lower risk of LBW or preterm birth than those of lower SES mothers (Table 2). However, inequalities in perinatal health are more pronounced in Montreal for both health indicators, before adjustement, and in the fully adjusted model (adjusted for income, education, marital status, age, parity, and sex of the child. For example, in Brussels, the risk of LBW for a newborn whose mother is less educated compared to a newborn whose mother is highly educated is, after adjustment, 1.20 (CI = 1.09–1.32) in Brussels and 1.67 (CI = 1.58–1.77) in Montreal. (Table 2). Furthermore, in Montreal, the relationship between SES and perinatal health in the general population follows a classic health gradient, with the risk of poorer perinatal health being inversely proportional to SES. In Brussels, however, this gradient is present for education but is less pronounced or even non-existent for income.

**Table 2:** Associations between SES and birth outcomes. Brussels vs Montreal

	**LBW**					
	**BRUSSELS **(N=97 844)			**MONTREAL **(N=214 589)		
	%	ORs (95% CI)	adjusted ORs*	%	ORs (95% CI)	adjusted ORs*
**Maternal education**						
High	4.08	1	1	3.66	1	1
Intermediate	4.81	1.18 (1.10-1.29)^a^	1.16 (1.05-1.26) ^b^	4.50	1.24 (1.17-1.30)^a^	1.26 (1.19-1.33)^a^
Low	4.80	1.19 (1.09-1.28)^a^	1.20 (1.09-1.32)^a^	5.86	1.64 (1.56-1.72)^a^	1.67 (1.58-1.77)^a^
**Income Quintile**						
Top	3.72	1	1	3.63	1	1
Fourth	4.59	1.24 (1.09-1.42)^b^	1.18 (1.03-1.35)^c^	4.25	1.18 (1.10-1.26)^a^	1.08 (1.01-1.16)^c^
Middle	4.83	1.31 (1.16-1.49)^a^	1.21 (1.05-1.38)^b^	4.58	1.27 (1.19-1.36)^a^	1.15 (1.07-1.23)^a^
Second	4.49	1.22 (1.07-1.37)^b^	1.15 (1.01-1.32)^c^	4.69	1.31 (1.22-1.40)^a^	1.14 (1.05-1.21)^a^
Bottom	4.66	1.26 (1.13-1.41)^a^	1.12 (0.98-1.27)	5.35	1.50 (1.40-1.60)^a^	1.29 (1.20-1.38)^a^
	**PRETERM**					
	**BRUSSELS **(N=95 490)			**MONTREAL **(N=214 587)		
	%	ORs (95% CI)	adjusted ORs*	%	ORs (95% CI)	adjusted ORs*
**Maternal education**						
High	4.68	1	1	4.77	1	1
Intermediate	5.38	1.16 (1.07-1.25)^a^	1.12 (1.03-1.22)^b^	5.95	1.26 (1.21-1.32)^a^	1.28 (1.22-1.34)^a^
Low	5.40	1.16 (1.08-1.25)^a^	1.14 (1.03-1.23)^b^	7.40	1.59 (1.52-1.67)^a^	1.60 (1.52-1.68)^a^
**Income**						
Top	4.46	1	1	5.10	1	1
Fourth	5.07	1.14 (1.01-1.29)^c^	1.11 (0.97-1.26)	5.57	1.09 (1.03-1.16)^b^	1.01 (0.95-1.07)
Midlle	5.15	1.16 (1.03-1.30)^c^	1.11 (0.97-1.25)	5.97	1.18 (1.11-1.25)^a^	1.07 (1.01-1.14)^c^
Second	5.08	1.15 (1.02-1.28)^c^	1.08 (0.95-1.23)	6.05	1.20 (1.13-1.27)^a^	1.04 (0.98-1.11)
Bottom	5.28	1.19 (1.08-1.32)^b^	1.07 (0.95-1.21)	6.50	1.29 (1.22-1.37)^a^	1.13 (1.06-1.20)^a^

^*^ORs adjusted for income, education, marital status, parity, mother’s age, and child’s sex

^a^ ≤ 0.001; ^b^ ≤ 0.01; ^c^ ≤ 0.05

#### Greater impact of SES among natives than immigrants

In both regions, the association between SES and perinatal health differs according to the mother’s birthplace (Tables 3 and 4). The impact of SES is stronger for mothers born in Belgium and Canada than for those born abroad. Among native mothers, all associations are significant, before and after adjusting for maternal and child characteristics. The magnitude of inequalities in perinatal health is, however, greater for mothers born in Canada than for those born in Belgium,except for the association between income and preterm birth (Table 3). The socioeconomic gradient in perinatal health is less marked among immigrant mothers than native mothers. This finding is more pronounced in Brussels than in Montreal, particularly for education, which is associated with pregnancy outcomes among immigrant in Montreal but not in Brussels (Table 4).

**Table 3:** Associations between SES and birth outcomes among natives-born women. Brussels vs Montreal

	**LBW**					
	**Belgian natives-born** (N=39 381)			**Canadian natives-born **(N=97 509)		
	%	ORs (95% CI)	Adjusted ORs*	%	ORs (95% CI)	Adjusted ORs*
**Maternal education**						
High	4.10	1	1	3.38	1	1
Intermediate	5.23	1.29 (1.15-1.44)^a^	1.23 (1.09-1.39)^a^	4.21	1.26 (1.16-1.36)^a^	1.25 (1.15-1.36)^a^
Low	6.27	1.56 (1.39-1.75)^a^	1.45 (1.23-1.66)^a^	6.20	1.89 (1.75-2.04)^a^	1.81 (1.65-1.98)^a^
**Income quintile**						
Top	3.89	1	1	3.31	1	1
Fourth	4.76	1.23 (1.05-1.44)^b^	1.16 (0.98-1.37)	4.01	1.22 (1.11-1.34)^a^	1.09 (0.98-1.20)
Middle	5.02	1.30 (1.11-1.52)^b^	1.16 (0.97-1.37)	4.62	1.42 (1.29-1.56)^a^	1.21 (1.09-1.34)^a^
Second	5.09	1.32 (1.13-1.55)^a^	1.22 (1.02-1.46)^c^	4.93	1.52 (1.38-1.67)^a^	1.23 (1.11-1.37)^a^
Bottom	5.59	1.46 (1.27-1.69)^a^	1.24 (1.05-1.48)^c^	5.82	1.81 (1.63-2.00)^a^	1.37 (1.22-1.53)^a^
	**PRETERM**					
	**Belgian natives-born** (N=38 670)			**Canadian natives-born **(N=97 509)		
	%	ORs (95% CI)	Adjusted ORs*	%	OR’s (95% CI)	Adjusted ORs*
**Maternal education**						
High	4.72	1	1	4.58	1	1
Intermediate	5.70	1.22 (1.09-1.35)^a^	1.15 (1.02-1.29)^c^	5.75	1.27 (1.19-1.36)^a^	1.28 (1.19-1.38)^a^
Low	6.34	1.36 (1.22-1.53)^a^	1.23 (1.07-1.41)^b^	7.70	1.74 (1.63-1.86)^a^	1.69 (1.57-1.83)^a^
**Income quintile**						
Top	4.55	1	1	4.86	1	1
Fourth	5.16	1.14 (0.98-1.32)	1.10 (0,94-1.28)	5.66	1.17 (1.08-1.27)^a^	1.05 (0.96-1.14)
Middle	5.20	1.15 (0.99-1.34)	1.07 (0.91-1.25)	5.88	1.22 (1.13-1.33)^a^	1.06 (0.97-1.15)
Second	5.58	1.24 (1.06-1.44)^c^	1.16 (0.97-1.37)	6.22	1.30 (1.19-1.41)^a^	1.07 (0.98-1.17)^c^
Bottom	5.84	1.30 (1.13-1.49)^a^	1.22 (1.03-1.44)^c^	6.87	1.44 (1.32-1.58)^a^	1.13 (1.02-1.25)^c^

^*^ORs adjusted for income, education, marital status, parity, mother’s age, and child’s sex

^a^ ≤ 0.001; ^b^ ≤ 0.01; ^c^ ≤ 0.05

**Table 4:** Associations between SES and birth outcomes among immigrants. Brussels vs Montreal

	**LBW**					
	**BRUSSELS **(N=55 333)			**MONTREAL **(N=112 463)		
	%	ORs (95% CI)	adjusted ORs*	%	ORs (95% CI)	adjusted ORs*
**Maternal education**						
High	4.08	1	1	3.94	1	1
Intermediate	4.50	1.11 (0.98-1.24)	1.05 (0.92-1.20)	4.80	1.23 (1.15-1.32)^a^	1.27 (1.18-1.37)^a^
Low	4.12	1.01 (0.90-1.13)	1.03 (0.89-1.18)	5.62	1.45 (1.35-1.56)^a^	1.57 (1.45-1.69)^a^
**Income Quintile**						
Top	3.29	1	1	4.13	1	1
Fourth	4.23	1.30 (1.01-1.67)^c^	1.22 (0.93-1.59)	4.54	1.10 (0.99-1.22)	1.04 (0.93-1.16)
Middle	4.59	1.41 (1.13-1.76)^b^	1.38 (1.09-1.75)^b^	4.61	1.12 (1.01-1.24)	1.05 (0.94-1.17)
Second	4.10	1.26 (1.02-1.55)^c^	1.28 (1.01-1.61)^c^	4.53	1.10 (0.99-1.21)	1.00 (0.90-1.10)
Bottom	4.28	1.31 (1.08-1.60)^b^	1.26 (1.02-1.57)^c^	5.18	1.27 (1.15-1.39)^a^	1.16 (1.04-1.27)^a^
	**PRETERM**					
	**BRUSSELS **(N=54 009)			**MONTREAL **(N=112 462)		
	%	ORs (95% CI)	adjusted ORs*	%	ORs (95% CI)	adjusted ORs*
**Maternal education**						
High	4.65	1	1	4.97	1	1
Intermediate	5.16	1.11 (1.00-1.24)	1.08 (0.95-1.23)	6.19	1.26 (1.18-1.34)^a^	1.28 (1.20-1.36)^a^
Low	4.97	1.07 (0.96-1.19)	1.08 (0.95-1.23)	7.21	1.48 (1.39-1.58)^a^	1.53 (1.43-1.64)^a^
**Income Quintile**						
Top	4.22	1	1	5.53	1	1
Fourth	4.92	1.17 (0.93-1.48)	1.17 (0.92-1.49)	5.52	0.99 (0.91-1.09)	0.93 (0.84-1.02)
Midlle	5.08	1.21 (0.99-1.48)	1.23 (0.98-1.52)	6.14	1.12 (1.02-1.22)^c^	1.05 (0.96-1.15)
Second	4.76	1.13 (0.94-1.37)	1.12 (0.91-1.38)	5.97	1.08 (0.99-1.18)	0.98 (0.90-1.08)
Bottom	5.03	1.20 (1.01-1.43)^c^	1.11 (0.91-1.35)	6.39	1.17 (1.07-1.26)^a^	1.07 (0.98-1.16)

^*^ORs adjusted for income, education, marital status, parity, mother’s age, and child’s sex

^a^ ≤ 0.001; ^b^ ≤ 0.01; ^c^ ≤ 0.05

## Discussion

The use of large-scale administrative databases has made it possible to assess inequalities in perinatal health in Brussels and Montreal. The analysis reveals similarities, but also notable differences between the two regions. First, inequalities in perinatal health are observed in both regions, but they are more pronounced in Montreal than in Brussels. Second, the association between SES and perinatal health varies according to the mother’s place of birth, with the impact of SES being greater for mothers born in Belgium or Canada than for those born abroad. However, the link between SES and perinatal health among immigrants is weaker in Brussels than in Montreal.

How can we explain the greater extent of inequalities in perinatal health in Montreal than in Brussels? Two complementary hypotheses will be discussed below: on the one hand, the existence of greater social inequalities in Montreal than in Brussels and, on the other hand, the lower vulnerability of immigrants with low SES in Brussels.

### Greater social inequalities in Montreal than in Brussels

The classic social gradient observed can be explained by stronger protective factors and lower health risk factors as one moves up the social ladder. The greater vulnerability of low-income mothers can be explained, for example, by insufficient income to acquire goods and services and by psychosocial consequences – namely social participation and the adverse consequences of social comparison. By comparing the two contexts, we can observe similar poverty rates: in 2016, if we consider a poverty threshold set at 50% of the median income, the poverty rate of the general population was at 8.3% in Belgium and 9.5% in Quebec [32, 33] and the child poverty rate was at 9.8% in Belgium and 9.7% in Quebec (under 16 years of age) [33, 34]. Poverty rates at the regional level are also similar – 18.9% in Brussels and 16.2% in Montreal [32, 33].

While the poverty rates are similar, the intensity of poverty, however, is greater in Quebec than in Belgium. The intensity of poverty is measured by the poverty gap, which is a relative estimate of the difference between the average or median income of low-income households and the relative poverty threshold. In Belgium, the poverty gap was at 21.6% in 2016, meaning the disposable income of poor people was on average 21.6% [35] below the poverty threshold. In Quebec, however, the poverty gap was at 30.3% (Source: Quebec Statistical Institute). This difference can be explained in particular by a lower replacement income for people outside the labour market in Quebec. This is the case for welfare: for a single person with no work income, was 11% below the relative poverty line (50% threshold) in Belgium and 64% in Quebec. Unemployment insurance benefits replace the income of the unemployed at a rate of 65% in Belgium and 55% in Quebec on average and for a longer period of time in Belgium than in Quebec [36]. Income inequality, as measured by the Gini index in 2017, is also more pronounced in Quebec (0.32) than in Belgium (0. 26) [32, 37].

All in all, if the proportion of low-income households is similar in both regions, the poor are relatively poorer in Quebec than in Belgium and live in a more unequal context. These differences between the two contexts could help explain the greater magnitude of inequalities in perinatal health in Montreal than in Brussels.

### Lower vulnerability of immigrants with low SES in Brussels

In both regions, the impact of SES is greater among mothers born in Belgium or Canada than among those born abroad. This difference according to the mother’s birthplace is more pronounced in Brussels than in Montreal, particularly with respect to maternal education. While in Montreal the risk of LBW or preterm birth progressively decreases as the education level increases, in Brussels, education is not at all associated with these risks in the case of immigrant mothers.

The weakness or absence of the socioeconomic gradient, mainly in terms of education level, pertaining to perinatal health among immigrants has also been highlighted in other studies [16–18, 38].

This finding is directly linked to the relatively low prevalence of LBW observed among some immigrant mothers with low SES: in the case of mothers with a low level of education, LBW is less prevalent among immigrants than among native women, particularly in Brussels. A study that compares immigrant and native mothers with equal SES confirms the lower vulnerability of immigrant women in Brussels to LBW and preterm delivery [23]. One explanation is the presence of protective factors that reduce the vulnerability of certain less educated immigrant mothers during pregnancy. For instance, the study conducted in Brussels showed that 60% of Brussels mothers of Maghrebi origin stayed at home during their pregnancy [18, 26]. Not being exposed to precarious working conditions during pregnancy could have a beneficial effect on the course of the pregnancy and the health of both mother and child, and contribute to the low risk of giving birth to LBW and preterm infants for Maghrebi mothers with a low SES. Another explanatory factor relates to lifestyle habits: tobacco and alcohol consumption is much less frequent among immigrants than among native women [39, 40].

The smaller social gradient in Brussels can be explained by a compositional effect: there are proportionally more immigrant mothers in low SES households in Brussels than in Montreal. In Brussels, 72% of very low-income households are immigrant households, as opposed to 56% in Montreal, and 69% of mothers with a low level of education are immigrants in Brussels compared to 54% in Montreal. The lower impact of income and education on perinatal health among immigrant mothers, particularly in Brussels, could help explain the lower inequalities in preterm birth and low birth weight in Brussels.

### Contributions and limitations of the study

The strengths of the study are related to the comparative approach adopted and the explanatory hypotheses put forward, as well as the use of rich databases in both contexts. Indeed, this study relies on population-based databases of births in Brussels and Montreal. Health data were coupled with socio-economic information from administrative databases*,* which made it possible to compare inequalities in perinatal health on the basis of two SES indicators.

We have chosen to compare contexts that are similar on several levels (urban character, general poverty rate, immigration rate, and perinatal health indicators in the general population), but have different income support policies. Beyond the comparison of the extent of health inequalities in the two regions, this approach, created a potential to compare differences in social and political conditions between the two study locations and discuss how these may contribute to differences in inequalities in perinatal health. The discussion explores two possible explanations of the more pronounced inequalitiesin perinatal health in Montreal than in Brussels. These explanations provide grounds for interrogation of public policies in each jurisdiction that may contribute to these differences. One of these is that the worse outcome for lower income mothers in Montreal could be explained by a greater level of ‘background’ socioeconomic inequity in Montreal and, in particular, more ‘intense’ states of poverty brought on by being further below a threshold poverty line. The very low generosity of social assistance in Quebec helps to explain the high intensity of household poverty in Quebec, compared to Belgium [41]. A lesson that emerges from our analysis is the value of considering several poverty indicators to better appreciate the situation of the poor in different contexts. Indeed, public health studies that look at the impact of social policies on health inequities only consider the poverty rate and analyse the correlation between this rate and inequities. Poverty gap is a complementary indicator to the poverty rate, which allows a better appreciation of the situation of the poorest. It also provides an indication of the inequality dimension as it reflects the extent to which the average income of the poor (irrespective of their number) is below that of the general population. Measures that can reduce the financial insecurity of the most vulnerable households are needed to reduce the intensity of poverty. This includes more generous policies for households outside the labour market or with very low labour market participation. This issue is even more critical in Quebec where social assistance policy and unemployment insurance benefits are less generous.

The other hypothesis is that immigrants with low SES in Brussels might be less vulnerable to poor perinatal health because they were protected by factors such as staying at home during pregnancy (rather than working in precarious employment) and lower levels of tobacco and alcohol consumption among immigrant mothers. As the paper reports, a socioeconomic gradient in perinatal health outcomes is well documented in established literature. Also, interestingly, this gradient tends to apply more among native-born mothers. Among foreign-born immigrant mothers, conversely, existing literature shows that that an association between SES and perinatal health outcomes is weak or absent. This paper adds to this literature by introducing a hypothesis linked to working conditions. Such a hypothesis is all the more plausible since it is known that the mother’s occupation has a significant impact on the risk of adverse pregnancy outcomes [12]. Workplace protection and safety measures that protect pregnant women from workplace hazards and harsh working conditions, would mitigate this risk.

While this analysis has many strengths, it is not without limitations. One limitation is inherent in all studies that seek to understand the causes of health inequalities. Indeed, these causes are multiple and interdependent. Perinatal health is no exception. The unavailability of certain information in our databases prevented us from exploring certain hypotheses further. For example, information on smoking habits would have made it possible to estimate the extent to which they contribute to differences in LBW between native and immigrant women in the two contexts. Data on smoking during pregnancy detailed by immigration proved difficult to obtain. Similarly, information on working conditions would also have been useful.

The difference in income data sources across both regions renders the comparison of income-related health less than ideal. In fact, the data on education level come from similar data sources in both regions and focus on the mother’s education, while the data on income are reported at the household level in Brussels and at the level of small geographic agglomerations in the Quebec context. While these data reflect the magnitude of inequalities as usually studied in the Quebec context, and can be used as a proxy for household income since the agglomerations are very small and fairly homogeneous in socio-economic terms, it would be relevant to also study inequalities at birth by household income in Quebec to compare possible differences in the magnitude of inequalities observed according to the type of data (geographic or individual). We are not aware of any studies of health inequalities at birth using income data at the individual level.

From a methodological standpoint, merging the two databases would have made it possible to go further in the analyses by directly comparing the health indicators observed in different groups according to socio-economic status and immigration or household composition. However, authorisations for such mergers remain difficult to obtain.

## Conclusion

Two regions with similar sociodemographic and perinatal indicators in the general population show significant differences in terms of inequalities in perinatal health. These results could be explained by the differing characteristics of low-income and immigrant households between the two contexts. Moreover, the analysis suggests that a comparison of immigration and poverty contexts, as well as the public policies related to these factors, can explain certain results in perinatal epidemiology. Future studies seeking to understand the mechanisms that lead to inequalities in perinatal health in different contexts should take this into account.

## Data Availability

Belgian data are available from the authors (MS and MDS) upon reasonable request and with permission of Commission for the Protection of Privacy (CPP). Canadian data are available at the Québec Inter-University Center for Social Statistics (QICSS).
